# The role of DPP6 dysregulation in neuropathology: from synaptic regulation to disease mechanisms

**DOI:** 10.3389/fncel.2025.1547495

**Published:** 2025-03-05

**Authors:** Xuan-Yan Ding, Jean de Dieu Habimana, Zhi-Yuan Li

**Affiliations:** ^1^Guangdong Provincial Key Laboratory of Stem Cell and Regenerative Medicine, Guangdong-Hong Kong Joint Laboratory for Stem Cell and Regenerative Medicine, Guangzhou Institutes of Biomedicine and Health, Chinese Academy of Sciences, Guangzhou, China; ^2^University of Chinese Academy of Sciences, Beijing, China

**Keywords:** DPP6, Kv4 channels, ion channel regulation, neurological diseases, cardiovascular disease

## Abstract

As a transmembrane protein, DPP6 modulates the function and properties of ion channels, playing a crucial role in various tissues, particularly in the brain. DPP6 interacts with potassium channel Kv4.2 (KCND2), enhancing its membrane expression and channel kinetics. Potassium ion channels are critical in progressing action potential formation and synaptic plasticity. Therefore, dysfunction of DPP6 can lead to significant health consequences. Abnormal DPP6 expression has been identified in several diseases, such as amyotrophic lateral sclerosis (ALS), autism spectrum disorder (ASD), spinal bulbar muscular atrophy (SBMA), and idiopathic ventricular fibrillation. Recent research has indicated a connection between DPP6 and Alzheimer’s disease as well. The most common symptoms resulting from DPP6 dysregulation are mental deficiency and muscle wastage. Notably, these symptoms do not always occur at the same time. Besides genetic factors, environmental factors also undoubtedly play a role in diseases related to DPP6 dysregulation. However, it remains unclear how the expression of DPP6 gets regulated. This review aims to summarize the associations between DPP6 and neurological diseases, offering insights as well as proposing hypotheses to elucidate the underlying mechanisms of DPP6 dysregulation.

## 1 Introduction

In mammals, Shal potassium channels (Kv4 channels), including Kv4.1, Kv4.2, and Kv4.3, are transmembrane voltage-gated ion channels characterized by a highly conserved structure. Compared to Kv1, Kv2, and Kv3 channels, Kv4 channels exhibit faster activation and inactivation kinetics, enabling precise temporal control of neuronal excitability. DPP6, a key modulator of Kv4 channels, is predominantly expressed in the brain, underscoring its critical role in regulating these ion channels and contributing significantly to processes such as chronic pain modulation and synaptic integration, which are closely associated with a range of neurological disorders (Birnbaum et al., 2004; Hu et al., 2006; Cai et al., 2004).

**Figure 1:**
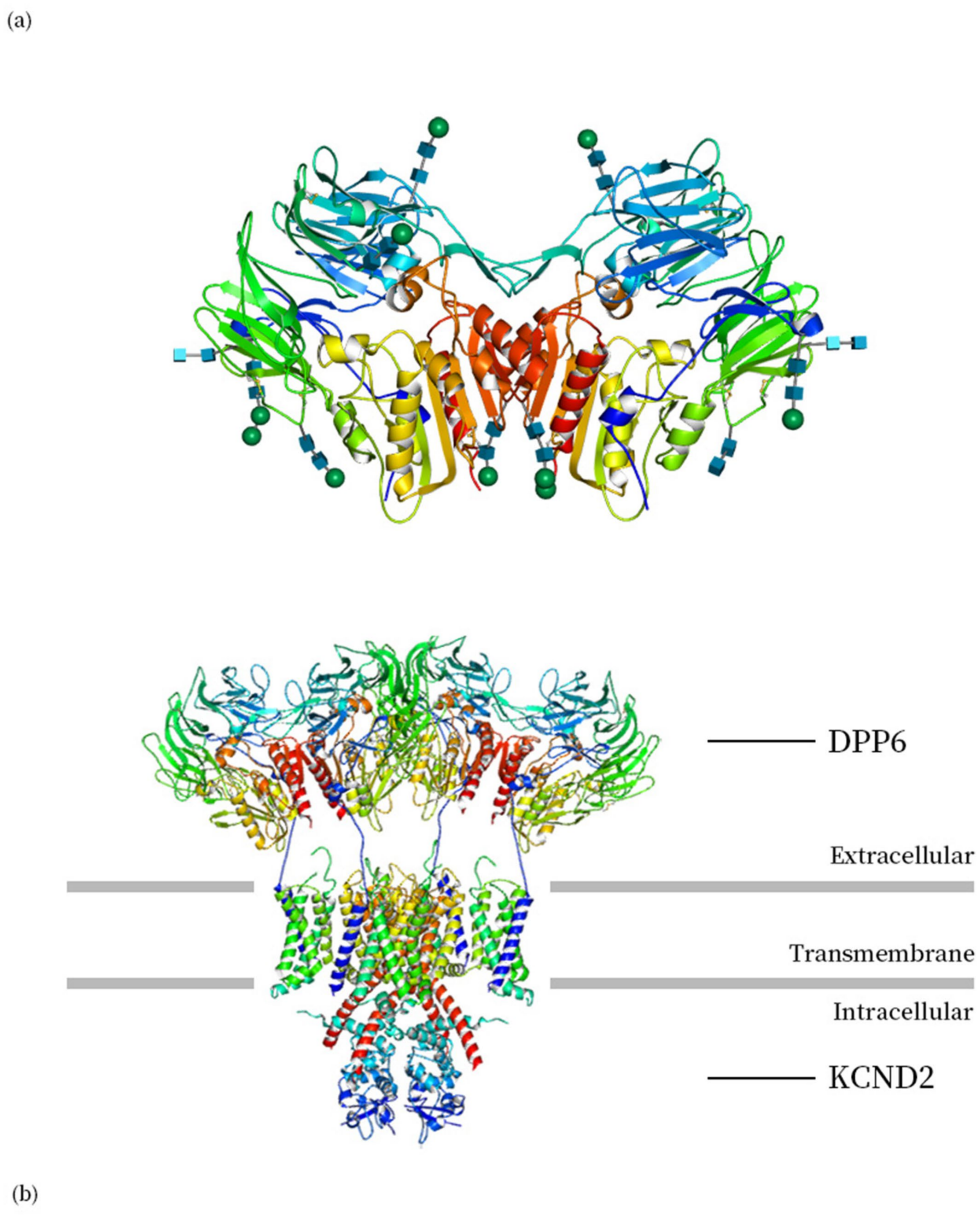
**(a)** The biological unit structure of DPP6 (DPP6 dimer, PDB ID: 1XFD) (Strop et al., 2004). **(b)** Structure of human Kv4.2-DPP6S complex (PDB ID: 7E8B) (Kise et al., 2021). Images are produced using Molmil, a WebGL Molecular Viewer developed by PDBj (Bekker et al., 2016, 2022; Kinjo et al., 2018).

The DPP6 protein (Dipeptidyl Peptidase Like 6, DPPX) was initially identified in bovine and rat brains as a transmembrane protein, also expressed in human and other mammalian species. The expression of DPP6 varies across tissues, with the highest levels observed in the brain (Wada et al., 1992; Kin et al., 2001). The intracellular N-terminal residue and the large extracellular domain are connected by the transmembrane domain, which functions as a dimer formed by two monomers, each monomer contains an eight-bladed *β*-propeller domain and an *α*/β hydrolase domain (Figure 1) (Strop et al., 2004).

The most notable feature of DPP6 is its interaction with the Shal type potassium ion channel Kv4. The presence or absence of the transmembrane helical region of DPP6, as an auxiliary subunit of Kv4.2, directly affects the channel efficiency of Kv4.2 (Boyden et al., 2016; Ye et al., 2022). By associating with Kv4 channels, DPP6 significantly enhances the expression of somatodendritic subthreshold A-type K+ currents (I_SA_), which are characterized by their rapid activation and inactivation within the subthreshold range of membrane potentials. These currents play a crucial role in shaping synaptic integration and plasticity, the fundamental mechanisms underlying learning and memory (Soh and Goldstein, 2008; Kaulin et al., 2009; Nadin and Pfaffinger, 2013). DPP6-KO mice showed impaired learning and memory abilities, as well as the appearance of aging markers in the brain (Lin et al., 2018, 2020). DPP6 is closely related to early neural development, and hippocampal neurons lacking DPP6 exhibit a decrease in dendritic and spine density (Lin et al., 2013). This suggests that DPP6 may be an important yet unexplored link in ion channel related diseases and neurodevelopmental disorders.

## 2 Symptoms related to DPP6 abnormalities

### 2.1 Intellectual disability and dementia

Anti-dipeptidyl-peptidase-like protein 6 encephalitis can lead to rapid progressive dementia (RPD), hippocampal atrophy, and a range of symptoms, including diarrhea and weight loss, hyperexcitability (such as myoclonus, tremor, spasticity, and muscle cramps), cognitive dysfunction (such as memory loss and executive dysfunction), and cerebellar ataxia, among others (Esechie et al., 2023). Loss of function of DPP6 is associated with autosomal dominant microcephaly, which can lead to varying degrees of intellectual disability (Liao et al., 2013).

Since dementia is a hallmark symptom of Alzheimer’s disease (AD), DPP6, as a protein closely associated with learning and memory, could be considered one of many candidate proteins involved in the pathogenesis of AD. In addition to exhibiting impaired hippocampal-dependent learning and memory abilities and reduced brain size, DPP6-KO mice display early-onset deposition of Alzheimer’s disease-related proteins, including amyloid *β*, *α*-synuclein, and phosphorylated tau. Moreover, aged DPP6-KO mice exhibit structural degeneration, characterized by reduced brain and hippocampal volume, a decreased total number of neurons, and increase in neuronal loss (Lin et al., 2022). Genetic variations in DPP6, including nonsense and frameshift variants as well as intronic SNPs, are associated with frontotemporal dementia (FTD). These mutations may contribute to reduced DPP6 protein levels, likely due to nonsense-mediated mRNA decay and haploinsufficiency (Cacace et al., 2019; Pottier et al., 2019).

Autism spectrum disorder (ASD) is a neurodevelopmental disorder characterized by deficits in social communication, as well as restricted interests and repetitive behaviors. Additionally, a significant number of individuals with ASD exhibit intellectual disabilities (Bartak and Rutter, 1976). Previous microarray and karyotype analyses have revealed DPP6 abnormalities in ASD patients, suggesting a potential link between DPP6 dysfunction and ASD (Marshall et al., 2008). Among patients with Gilles de la Tourette syndrome (TS) who also have neurodevelopmental disorders, clinical studies have shown that 2.9 to 20% of patients have comorbid ASD, which is higher than the general incidence (Huisman-van Dijk et al., 2016). TS is mainly manifested as tic symptoms, which are believed to be related to dopaminergic regulation of the central nervous system, and DPP6 regulates Kv4 properties, providing a possibly related influencing factor at the molecular level (Robertson et al., 2017). Although there are suspicions that this high probability is due to similar symptoms interfering with diagnosis, mutations lead to downregulating DPP6 expression have indeed been occurred in some TS patients (Darrow et al., 2017; Prontera et al., 2014).

Haloperidol is one of the two drugs approved by the FDA for the treatment of Tourette’s syndrome, and symptoms are significantly reduced in children with autism who receive treatment with Haloperidol (Anderson et al., 1989; Remington et al., 2001; Campbell et al., 1978). Haloperidol is a first-generation typical antipsychotic drug widely used worldwide, which works by blocking dopamine D2 receptors in the brain (Seeman and Tallerico, 1998). It has been reported that long-term use of Haloperidol leading to increased expression of Kv4.3 channels may be the reason for the long-term changes in the excitability of dopaminergic neurons (Hahn et al., 2003). However, long-term use of haloperidol may lead to adverse reactions, including delayed onset of motor disorders such as tardive dyskinesia (TD). In a mouse model, long-term treatment of haloperidol resulted in decreased DPP6 expression in the brain, which is considered one of the possible causes of this adverse reaction (Tanaka et al., 2013). This reduction in DPP6 expression might be linked to the up regulation of Kv4 channels, potentially triggering a feedback regulatory mechanism. While the precise relationship between haloperidol and DPP6 remains unclear, and no direct evidence of binding exists, further research is needed to explore whether DPP6 plays a role in haloperidol’s effects on the central nervous system.

### 2.2 Motion and skeletal muscles

Amyotrophic Lateral Sclerosis (ALS) is a neurodegenerative disease primarily affecting motor neurons, characterized by muscle atrophy and loss of function. One in ten patients is a familial case. Several previous GWAS studies in European populations have suggested an association between DPP6 mutations and ALS. However, subsequent similar studies were unable to replicate this result in different populations (Van Es et al., 2008; Fogh et al., 2011; Daoud et al., 2010). Meanwhile, motor neurons reprogrammed from somatic cells of ALS patients using induced pluripotent stem cell (iPSC) models revealed a significant downregulation of DPP6 expression, as demonstrated by qPCR analysis (Sareen et al., 2013).

Similar to ALS, motor neurons in Spinal and Bulbar Muscular Atrophy (SBMA) undergo degenerative death, while Multiple Sclerosis (MS) mainly involves the central nervous system (brain and spinal cord) and has relatively less effect on lower motor neurons (Sahashi et al., 2015). However, these three diseases ultimately lead to motor disorders and muscle atrophy. A study on multiple sclerosis in the Nordic population showed that DPP6 levels were elevated in PBMCs of PrMS patients, although DPP6 is known to be mainly expressed in the brain and spinal cord, and DPP6 downregulation was observed in spinal motor neurons induced by SBMA iPSC differentiation (Brambilla, 2012; Sheila, 2019).

The changes in the expression levels of DPP6 in motor neurons, induced by reprogramming patient-derived cells, support the association of DPP6 with the aforementioned diseases. However, several GWAS studies have produced conflicting results, which may be attributed to the small sample size of rare diseases, the complex involvement of multi-gene interactions in ALS, or environmental factors.

Existing research has mostly focused on the role of DPP6 as a Kv4 helper subunit in the nervous system and heart, while research into its role in skeletal muscle and other tissues is still insufficient. The elevated expression level of DPP6 in PBMCs of PrMS patients suggests that pathological conditions may lead to high expression of DPP6 in other locations, or this ectopic expression itself may be one of the pathogenic factors. In addition to the aforementioned muscle atrophy-related diseases, short stature and low weight are also widely present in patients with DPP6 dysfunction, suggesting that DPP6 may have cross-system functions (Liao et al., 2013).

### 2.3 Ventricular fibrillation

Cardiomyocytes rely on complex ion channel processes on the membrane to achieve their functions. Among these, Kv4 channels primarily regulate the transient outward potassium current (I_to_), which plays a crucial role in the early repolarization of the cardiac action potential and serves as a key determinant of action potential morphology. DPP6, as an auxiliary subunit of Kv4, enhances its surface expression and potently accelerates its gating kinetics, thereby ensuring the normal activity of cardiomyocytes. Among heart-related diseases, Sudden Cardiac Death (SCD) has a high incidence rate and a large social burden (Stecker et al., 2014). Idiopathic vascular fibrosis (IVF) is one of the causes of SCD, and DPP6 mutation is a pathogenic factor of IVF (Viskin, 1990).

A typical risk mutation occurred in a multi-generational family in the Netherlands. In patients carrying the corresponding 7q36 mutation, the mRNA expression level of DPP6 is 22 times higher than that of the control group. This overexpression increases Ito and increases the probability of sudden cardiac death in patients. However, no partial mutations in the DPP6 exon were detected, indicating that this regulatory mutation may occur outside of the exon (Alders et al., 2009; Postema et al., 2011). In contrast to the increase in Ito when DPP6 is overexpressed, a decrease in Ito density is observed when DPP6 is knocked down (Xiao et al., 2013).

This disease risk is believed to be related to Purkinje Fibers (PF). In PF, overexpression of DPP6 markedly increased Ito density and enhanced sensitivity to tetraethylamine (TEA). Previous studies have shown that the sensitivity of Kv4 channels to TEA depends on the presence of DPP6 (Colinas et al., 2008). However, similar Ito enhancement and TEA sensitivity were not observed when DPP6 was overexpressed in ventricular myocytes (VM) (Xiao et al., 2013). This difference may be attributed to variations in cellular structure, Kv4 channels, and the abundance of other regulatory factors in PF compared to VM, which may facilitate a more pronounced regulatory role of DPP6 in PF.

In the heart, NaV1.5, encoded by the SCN5A gene, is the primary sodium channel responsible for generating the sodium current (I_Na_), driving the rapid upstroke of the cardiac action potential (AP). While most of the I_Na_ (peak I_Na_) is rapidly inactivated, a small fraction (I_Na_ late) persists throughout the AP plateau phase. Beyond its role as an auxiliary subunit of Ito channels, DPP6 has been shown to regulate NaV1.5 by reducing both peak I_Na_ and late I_Na_ in CHO cells. Furthermore, prolonged QT/QU intervals on an electrocardiogram (ECG) indicate delayed cardiac repolarization and an increased risk of arrhythmias; DPP6 mutations may contribute to these abnormalities, thereby increasing arrhythmia susceptibility (Rossetti et al., 2022).

Therefore, preventing sudden death is one of the important application directions of DPP6 research. In DPP6 haplotype carriers, primary prevention by implanting an ICD (implantable cardiac defibrillator) has been shown to significantly reduce the rate of sudden death in patients who have not undergone IVF. Meanwhile, the research results indicate that the mortality rate and arrhythmia recurrence rate of male patients are still relatively high, therefore further adjustments and optimization of treatment strategies are needed. In addition, infections and unnecessary electric shocks caused by ICD implantation remain pressing concerns. However, studies have suggested that median survival time of patients carrying the DPP6 risk haplotype has significantly increased compared to earlier estimates, which indicates that cascade screening, early diagnosis, and ICD treatment have contributed to mortality reduction (Bergeman et al., 2023; Elmonzer, 2022). Future research could combine genetic screening to develop more accurate risk prediction models for personalized interventions.

## 3 Future directions

In summary, due to the regulatory role of DPP6 on ion channels, mutations of DPP6 are associated with abnormal discharges of excitable cells, which may further disrupt cellular functions and potentially lead to effects at the individual level. As mentioned earlier, DPP6-related diseases and their characteristics are shown in Table 1. It is evident that these conditions involve highly diverse pathogenic mechanisms. However, the symptoms associated with DPP6 mutations mentioned above typically do not manifest simultaneously in individuals carrying the mutation. The interaction between DPP6 and other regulatory factors, such as KCNIP2, FLNC, within different cells plays a crucial role in its functional modulation.

**Table 1:** DPP6 related diseases and their characterization.

Similar symptoms		Related disease	References
Affected mental abilities and social skills	Forgetfulness/memory loss Speech and language difficulties Motor abnormalities	Dementia	Lin et al. (2020) and Cacace et al. (2019)
		Alzheimer’s disease	Lin et al. (2020, 2022)
		Parkinson’s disease	Li et al. (2024)
		Microcephaly and intellectual difficulty	Liao et al. (2013)
		Autism spectrum disorder (ASD)	Marshall et al. (2008)
Abnormal muscle movements (including myocardial and skeletal muscles)	Uncontrollable movement	Gilles de la Tourette syndrome (GTS)	Prontera et al. (2014)
		Neuroleptic-induced tardive dyskinesia (TD)	Tanaka et al. (2013)
	Muscle weakness and atrophy	Amyotrophic lateral sclerosis (ALS)	Van Es et al. (2008) and Sareen et al. (2013)
		Spinal and Bulbar Muscular Atrophy (SBMA)	Sahashi et al. (2015) and Sheila (2019)
	Abnormal myocardial motion	Ventricular fibrillation	Postema et al. (2011) and Bergeman et al. (2023)

A recent study has revealed that DPP6 is one of the factors contributing to cognitive decline in Parkinson’s disease (Li et al., 2024). Given the strong association of DPP6 with Alzheimer’s disease and frontotemporal dementia, as well as the shared features among ALS, MS, and SBMA, it is worth considering whether these complex neurodegenerative diseases share a common molecular mechanism potentially linked to DPP6 dysfunction.

Interestingly, a study on organ-derived extracellular vesicles in mouse serum showed that DPP6, as an extracellular vesicle protein, exhibits 100% specificity to the brain, meaning it was exclusively detected in brain-derived extracellular vesicles with no peptides found in vesicles from other organs. Furthermore, the content of this protein in the extracellular vesicles of elderly mice is lower than that in young mice (Abdelmohsen et al., 2023). These extracellular vesicles can transport their contents across the blood–brain barrier, suggesting that the DPP6 content in serum extracellular vesicles could serve as a potential biomarker for dementia. This method does not require cerebrospinal fluid and can be easily detected through collecting blood. Another study showed that mutations in the DPP6 gene are more prevalent in the plasma exosomes of patients with neuroblastoma, but its precise role in this disease remains unclear (Degli Esposti et al., 2021).

Currently, research on DPP6 mainly focuses on high-expression tissues, and the study of its effects on other tissue cells is still minimal. However, previous reports have shown that DPP6 regulation is equally important outside of the heart and brain. For example, DPP6 could be one of the genes associated with lipid metabolism regulation, as indicated by lipid mass spectrometry analysis. Alterations in lipid metabolism can lead to cellular apoptosis or necrosis, potentially affecting tissues such as skeletal muscle (Martin et al., 2012).

Considering the role of DPP6 in different tissues and even in extracellular vehicles, it should be afforded necessary attention. Stem cell related technologies can be applied to expand research on DPP6. Specifically, DPP6 mutant patient derived cells and normal human derived cells (e.g., urine epithelial cells) can be collected and reprogrammed to induce differentiation *in vitro* to obtain different types of cell models including skeletal muscle cells, cardiomyocytes, and nerve cells.

Different samples can be compared using transcriptomics, proteomics, and other methods to analyze the expression and regulation of DPP6 in muscle tissue. Different tissue-specific DPP6 gene knockout models using Cre-loxP and other systems can be utilized to investigate their roles in muscle development and pathological processes at the individual level.

As described in the section on the risk of ventricular fibrillation associated with DPP6 mutations, the effects of DPP6 overexpression are more pronounced in PF than in VM. This suggests the potential existence of a compensatory or feedback inhibition mechanism regulating DPP6 expression in VM. Unraveling this mechanism could provide deeper insights into the pathophysiology of DPP6-related cardiac disorders and inform potential treatment strategies. Similarly, the directed differentiation of iPSCs derived from SBMA, IVF, and ASD patients carrying DPP6 mutations could facilitate the investigation of key cell types, including neurons, PF, and muscle cells. By analyzing differences in gene expression profiles, electrophysiological properties, and other cellular characteristics, we may gain a better understanding of how distinct DPP6 mutations and expression levels contribute to disease pathology, potentially identifying novel therapeutic targets.

While DPP6 is well established as a modulator of ion channels in specific neuronal populations, its functions in non-neuronal cells remain largely unexplored.

In future research, single-cell RNA sequencing or conditional knockout models could be utilized to determine whether DPP6 plays a role in glial cell function, thereby expanding our understanding of its physiological roles beyond synaptic regulation. In addition, the diagnosis of neurological diseases is an important direction for the application of DPP6. The diagnosis of neurological diseases such as AD and PD is difficult due to the difficulty and high risk of extracting cerebrospinal fluid for biomarker detection. When behavioral abnormalities are observed, the damage to the nervous system is usually irreversible. Based on the differential levels of DPP6 in brain derived extracellular vesicles in peripheral blood, it is possible to explore its potential as a biomarker for neurological diseases through clinical research.
